# Comparison of the Level and Mechanisms of Toxicity of Carbon Nanotubes, Carbon Nanofibers, and Silicon Nanotubes in Bioassay with Four Marine Microalgae

**DOI:** 10.3390/nano10030485

**Published:** 2020-03-08

**Authors:** Konstantin Pikula, Vladimir Chaika, Alexander Zakharenko, Zhanna Markina, Aleksey Vedyagin, Vladimir Kuznetsov, Alexander Gusev, Soojin Park, Kirill Golokhvast

**Affiliations:** 1 Far Eastern Federal University, Sukhanova 8, 690950 Vladivostok, Russian; chayka.vv@dvfu.ru (V.C.); zakharenko.am@dvfu.ru (A.Z.); markina.zhv@dvfu.ru (Z.M.); golokhvast.ks@dvfu.ru (K.G.); 2 National Scientific Center of Marine Biology, Far Eastern Branch, Russian Academy of Sciences, Palchevsky 17, 690041 Vladivostok, Russian; 3 Boreskov Institute of Catalysis SB RAS, Lavrentiev Ave. 5, 630090 Novosibirsk, Russian; vedyagin@catalysis.ru (A.V.); kuznet@catalysis.ru (V.K.); 4 Tambov State University named after G.R. Derzhavin, Internatsionalnaya 33, 392000 Tambov, Russian; nanosecurity@mail.ru; 5 National University of Science and Technology «MISIS», Leninskiy prospekt 4, 119049 Moscow, Russian; 6 Inha University, 100 Inharo, Nam-gu, Incheon 22212, Korea; 7 Pacific Geographical Institute, Far Eastern Branch of the Russian Academy of Sciences, Radio 7, 690041 Vladivostok, Russian; 8 Vavilov All-Russian Institute of Plant Genetic Resources, B.Morskaya 42-44, 190000 Saint-Petersburg, Russian

**Keywords:** carbon nanotubes, microalgae, mode of action, nanofibers, silica nanotubes, toxicity

## Abstract

Nanoparticles (NPs) have various applications in medicine, cosmetics, optics, catalysis, environmental purification, and other areas nowadays. With an increasing annual production of NPs, the risks of their harmful influence to the environment and human health is rising. Currently, our knowledge about the mechanisms of interaction between NPs and living organisms is limited. Additionally, poor understanding of how physical and chemical characteristic and different conditions influence the toxicity of NPs restrict our attempts to develop the standards and regulations which might allow us to maintain safe living conditions. The marine species and their habitat environment are under continuous stress due to anthropogenic activities which result in the appearance of NPs in the aquatic environment. Our study aimed to evaluate and compare biochemical effects caused by the influence of different types of carbon nanotubes, carbon nanofibers, and silica nanotubes on four marine microalgae species. We evaluated the changes in growth-rate, esterase activity, membrane polarization, and size changes of microalgae cells using flow cytometry method. Our results demonstrated that toxic effects caused by the carbon nanotubes strongly correlated with the content of heavy metal impurities in the NPs. More hydrophobic carbon NPs with less ordered structure had a higher impact on the red microalgae *P. purpureum* because of higher adherence between the particles and mucous covering of the algae. Silica NPs caused significant inhibition of microalgae growth-rate predominantly produced by mechanical influence.

## 1. Introduction

The growing industry of nanotechnology inevitably results in an increase of risks associated with the nanomaterials (NMs) and nanoparticles (NPs) to affect living organisms including humans. Carbon- and silica-based NPs could be highlighted among the variety of engineered NPs [1,2]. Due to its unique properties [3,4], carbon nanotubes and nanofibers are one of the most promising classes of NMs, which currently are in the growth phase of large-scale bulk production [5]. Carbon nanotubes were successfully used in electronics [6], catalysis [7,8], environmental purification [9,10], biomedicine [11,12], and other fields. Carbon nanofibers have an application in energy storage [13,14], electronics [15], catalysis [16,17], biosensing [18], etc. Silica NPs are mostly valued by their mesoporous properties [19]. Silica nanotubes, in particular, are widely used in drug delivery [20,21], catalysis [22,23,24], and biosensing [25].

The consumer products, large-scale manufacturers producing NMs, synthesizing, and research laboratories could be highlighted among the main sources leading to the release of NPs in the environment [26,27]. Despite intense interest in the problem of interaction between NPs and organisms and a relatively big amount of experimental data, the mechanisms of toxic action for NPs are still not fully clear [28,29,30]. Hence, the risk evaluation of nano-bio interaction, regulation, and development of the standards for safe production and utilization of NMs and NPs has become one of the high-priority problems in nanotechnology and nanotoxicology [31,32].

The difficulty of risk assessment in nanotoxicology is complicated by the variety of factors that could significantly change the properties and, therefore, the toxicity of NPs. Moreover, much uncertainty still exists about the relation between different parameters such as size, form, surface area, zeta potential of NPs, protein corona formation, and transformation of NPs inside of organisms and in the environment or combinations of these parameters and toxic properties of NPs [28,33,34,35]. NPs appear in the aquatic environment by surface wash, atmospheric sedimentation, and direct spills occurring during their synthesis, application, and utilization. The entrance of synthetic nanofibers, NPs, and NMs to water bodies has been reported in earlier studies [36,37,38,39]. Aquatic organisms such as microalgae species are known as reliable research objects in toxicology, and they are one of the most commonly used organisms in aquatic toxicity assessment of NPs [40,41,42,43]. Previous research has established that carbon nanotubes could cause changes in biochemical composition of microalgae [44], could attach to or penetrate algal cells [45], and the NPs may become involved in the transfer between the levels of food chain [46]. In contrast to carbon nanotubes, there is much less information about effects of carbon nanofibers and silica nanotubes on microalgae [41].

The aim of this study was to evaluate and compare the toxic level and biochemical effects caused by the influence of different types of carbon nanotubes, carbon nanofibers, and silica nanotubes on four marine microalgae species, namely *Attheya ussuriensis* (Bacillariophyceae), *Chaetoceros muelleri* (Bacillariophyceae), *Heterosigma akashiwo* (Raphidophyceae), and *Porphyridium purpureum* (Rhodophyceae).

## 2. Materials and Methods

### 2.1. Nanoparticles

In this research, we used two types of multiwalled carbon nanotubes (CNT-1, CNT-2) [47], two types of carbon nanofibers (CNF-1, CNF-2) [47], and two types of silica nanotubes (SNT-1, SNT-2) [48].

Carbon nanotubes and nanofibers were synthesized and characterized in the Boreskov Institute of Catalysis (Novosibirsk, Russia) [47]. The structural features of carbon NPs ware assessed by Raman spectroscopy in our earlier report [41]. The length of carbon nanotubes was hundreds of times larger than the diameter and in water suspension; the particles could cohere into the spheres up to tens of micrometers in diameter. The toxicity of these NP samples was previously evaluated on mice [49], rats [50,51], human cell lines [52], and microalgae *Heterosigma akashiwo* [41].

Silicon nanotubes were kindly provided by the Department of Chemistry, Inha University Republic of Korea [48]. The samples had a significantly lower ratio of length to diameter compared to carbon nanotubes.

Characteristics of NPs used in this research are represented in Table 1.

### 2.2. Microalgae Cultures

Microalgal cultures were provided by The Resource Collection *Marine biobank* of the National Scientific Center of Marine Biology, Far Eastern Branch of the Russian Academy of Sciences (NSCMB FEB RAS). The toxicity bioassay of NPs was carried out on four marine microalgae: two types of diatom species *Attheya ussuriensis* [53] and *Chaetoceros muelleri* [54], a raphidophyte *Heterosigma akashiwo* [55], and a red algae *Porphyridium purpureum* [56]. Culturing of microalgae and toxicity test conditions were maintained in accordance with the OECD Guidelines for the Testing of Chemicals, STest No.201 [57] with minor modifications as previously described [58,59].

The microalgae species were selected based on its abundance among the microalgae of the Sea of Japan [60], and its suitability as test objects in ecotoxicology [59,61,62,63]. Earlier, we confirmed the sensitivity of used microalgae species as test organisms with common reference toxicant potassium dichromate [58].

### 2.3. Bioassay

The samples of NPs were added to filtered seawater to obtain the working suspensions with a concentration of 1000 mg/L. Before each series of bioassays, the working suspensions of NPs were sonicated with ultrasound homogenizer Bandelin Sonopuls GM 3100 (Bandelin Electronic GmbH & Co. KG, Berlin, Germany) using maximal intensity for 30 min.

The exposition of microalgae cells to the suspensions of NPs was carried out in 24-well plates. Each well was filled with 2 mL of microalgae cell aliquot and the corresponding volume of the working suspension to obtain the final concentrations 1, 10, and 100 mg/L. The filtered seawater without NPs was added to the control group. The exposure of each used concentration and control group was performed in four biological replicates.

### 2.4. Flow Cytometry

Microalgae cell counting and registration of morphological and biochemical changes during the experiment were carried out with flow cytometer CytoFLEX (Beckman Coulter, Indianapolis, IN, USA) with the software package CytExpert v.2.0. The changes of microalgae cells after exposure to NPs were evaluated using specific fluorescent dyes. Microalgae growth-rate inhibition was determined by staining with propidium iodide (PI) according to the standard bioassay protocol [64]. Esterase activity of microalgae exposed to the NPs was evaluated using non-fluorescent lipophilic dye fluorescein diacetate (FDA) [65,66]. Membrane potential of microalgae cells was assessed by a lipophilic, positively charged fluorescent dye 3,3′-dihexyloxacarbocyanine iodide (DiOC_6_) [67,68]. To determine the size of microalgae cells, a size calibration kit, batch F13838 (Molecular probes, Eugene, OR, USA) with the certified size distribution of 1, 2, 4, 6, 10, 15 μm was used for the forward scatter emission channel. The emission channels were selected according to the maximum emission of the dyes, provided by the manufacturer (Molecular Probes, Eugene, OR, USA). The blue laser (488 nm) of the CytoFLEX flow cytometer was chosen as a source of excitation light. The endpoints of toxicity used in this work and the parameters of their registration are listed in Table 2. Each sample was measured at a flow rate of 100 μL/min for 30 s.

Prior to the assessment of growth-inhibition, esterase activity, and membrane potential of each microalgae species, we made a series of preliminary measurements to determine the optimal concentration of fluorescent dyes and the optimal duration of staining as described in our previous report [59]. The registration time for used endpoints was selected according to the standard methods commonly used to assess toxicity of a test substance in an aqueous system with microalgae model organisms [69,70,71,72].

Growth-rate inhibition and changes in the size of microalgae cells should be estimated as a benchmark of direct cytotoxic effects or as an indicator of mortality. The 96 h and 7 days half maximal effective concentration (EC_50_) are one of the most common values used for evaluation cytotoxic effects in macroalgae bioassay [63,73].

Esterase activity and membrane potential changes can indicate either the preliminary stage of toxic action or display the adaptational ability of organisms to influence toxic substances [74,75]. Changes of microalgae esterase activity are mostly caused by the deficiency of enzyme action or by disruption of membrane integrity, and it can be evaluated as a sensitive endpoint of algal sublethal toxicity [65,66]. The 3 h and 24 h registration points were chosen to detect possible early metabolic response of the algae over short exposure periods and dynamic change of that response, respectfully [71]. Reduction of membrane potential (depolarization) can be accompanied by changes of membrane elasticity, loss of lipid microdomains, and changes of ion permeability [76]. Integrity and normal operation of membranes are vital parameters for organisms as they provide barriers and transportation functions. The 6 h and 24 h registration points were chosen to detect changes in membrane potential of microalgae cells [72].

### 2.5. Microscopy

Morphological changes of microalgae cells were observed and captured by optical microscope Axio Observer A1 (Carl Zeiss, Oberkochen, Germany).

### 2.6. Statistical Analysis

Statistical analyses were performed using the software package GraphPad Prism 7.04 (GraphPad Software, San Diego, CA, USA). The one-way ANOVA test was used for analysis. A value of *p* ≤ 0.05 was considered statistically significant.

## 3. Results

For all the samples of NPs, we calculated EC_50_ concentrations of microalgae growth-rate, FDA fluorescence intensity (esterase activity), and DiOC_6_ (membrane potential) fluorescence intensity compared to control. The calculated mean EC_50_ concentrations and 95% confidence limits are given in Table 3.

**Figure 1:**
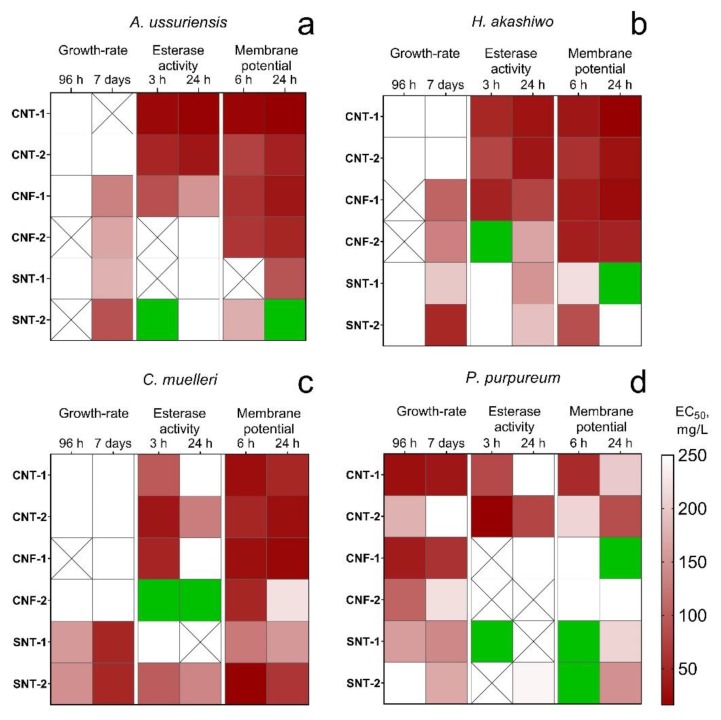
The heatmap of changes in microalgae state after the treatment with the NPs: (**a**) *A. ussuriensis*; (**b**) *H. akashiwo*; (**c**) *C. muelleri*; (**d**) *P. purpureum*; white square, the calculated EC_50_ was higher than 250 mg/L; crossed square, measured endpoint was not observed; green square, the influence of the NPs caused the stimulation of the measured endpoint.

For visualization of calculated data (Table 3) and analysis of dynamic changes of microalgae cells, we created a heatmap (Figure 1).

Silica nanotubes SNT-1 and SNT-2 demonstrated the most pronounced influence on the microalgae growth rate. The level of toxicity increased for all microalgae species after seven days of exposure except for the samples SNT-1 and SNT-2 after 96 h of the treatment affected growth-rate only for *C. muelleri* and *P. purpureum*.

Carbon nanotubes CNT-1 and CNT-2 had almost no influence on the growth rate of *A. ussuriensis*, *C. muelleri,* and *H. akashiwo* both after 96 h (acute toxicity) and seven days (chronic toxicity) of the treatment. Carbon nanofibers CNF-1 and CNF-2 also did not reveal any significant influence on the growth rate of these three species in acute toxicity assessment but chronic toxicity and growth-rate inhibition was detected for *A. ussuriensis* and *H. akashiwo*. Moreover, all the carbon NPs caused significant inhibition of esterase activity and depolarization of membranes for *A. ussuriensis*, *H. akashiwo*, and *C. muelleri*. However, the nanofiber sample CNF-2 did not affect the esterase activity of diatomic algae *A. ussuriensis* and stimulated esterase activity of diatomic algae *C. muelleri*.

The toxicological profile of the red algae *P. purpureum* significantly differed from three other microalgae species. *P. purpureum* was the only species responded by growth-rate inhibition to the influence of carbon NPs. At the same time, the red algae had the lowest changes in esterase activity and membrane potential.

**Figure 2:**
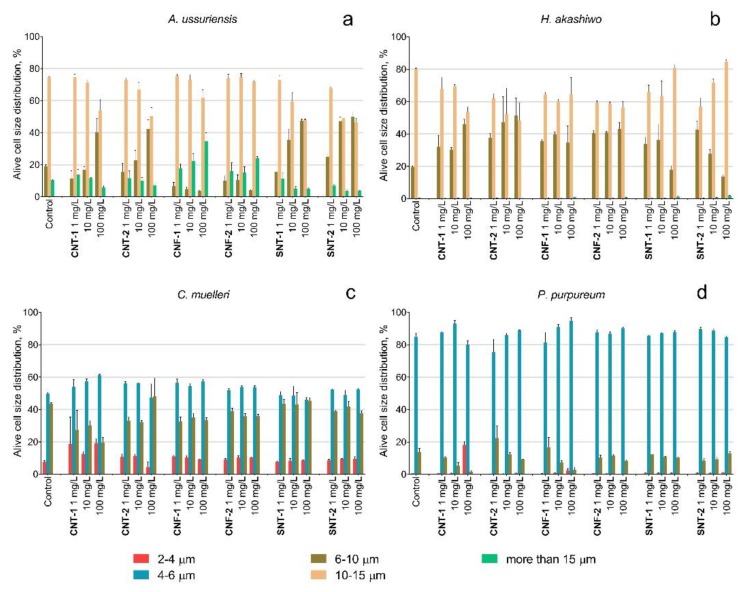
The changes of size distribution of microalgae cells after 96 h of exposure to NPs: (**a**) *A. ussuriensis*; (**b**) *H. akashiwo*; (**c**) *C. muelleri*; (**d**) *P. purpureum*.

The data of flow cytometry analysis indicated the changes in the size of microalgae cells after the treatment of NPs as demonstrated in Figure 2.

A decrease in the cell size was detected for diatomic algae *A. ussuriensis* after the treatment with the samples CNT-1 and CNT-2 at the concentration of 100 mg/L and after the treatment with silica nanotubes at the concentrations of 10 and 100 mg/L (Figure 2a). The carbon nanofibers CNF-1 caused enlargement of the cells of *A. ussuriensis* at the concentrations of 100 mg/L. The cells of *H. akashiwo* responded with a decreased cell size after of the interaction with all tested types of NPs (Figure 2b). The cell sizes of *C. muelleri* and *P. purpureum* slightly decreased after the treatment with the sample CNT-1 (Figure 2c,d).

**Figure 3:**
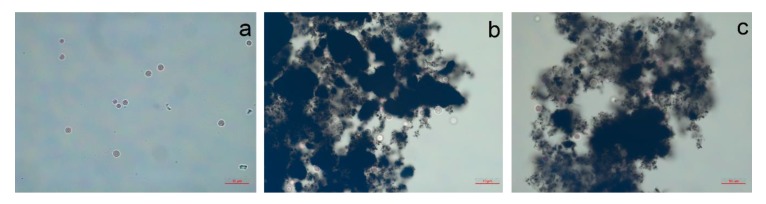
The red algae *P. purpureum* after 96 h of treatment with carbon nanotubes at concentration of 100 mg/L: (**a**) control; (**b**) CNT-1; (**c**) CNT-2.

**Figure 4:**
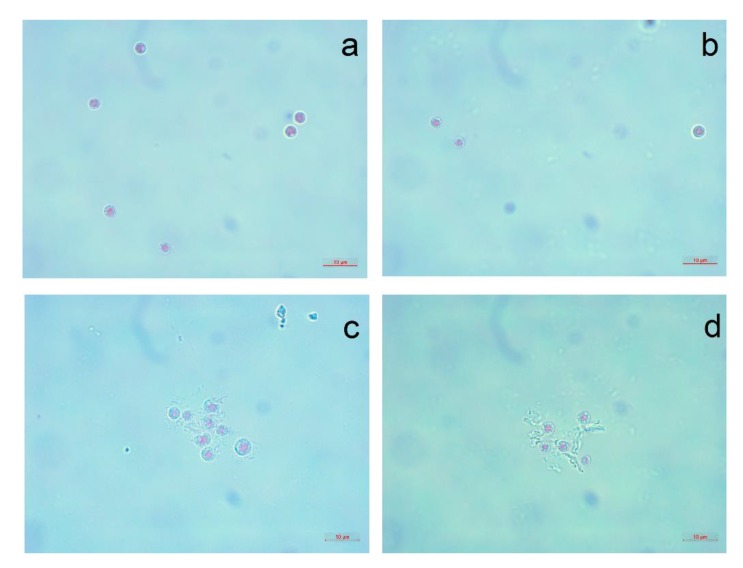
The red algae *P. purpureum* after 96 h of treatment with carbon nanofibers and silica nanotubes at concentration of 100 mg/L: (**a**) CNF-1; (**b**) CNF-2; (**c**) SNT-1; (**d**) SNT-2.

The visual observation of *P. purpureum* after seven days of exposure to the NPs is presented in Figure 3 and Figure 4.

It should be noted that despite a visible dissimilarity in the sensitivity and responses of different microalgae cells to the treatment of the NPs, we highlighted general mechanisms of action for assessed samples. In the next section, we discuss the principal findings of this investigation.

## 4. Discussion

### 4.1. Carbon Nanotubes and Nanofibers

One of the most peculiar observations that we can highlight from experimental data is a relatively high sensitivity of the red microalgae *P. purpureum* to the samples of carbon NPs and a lower sensitivity to the silica nanotubes, although the other three microalgae species had diametrically opposite responses (Table 3, Figure 1). The probable reason for such differences is a highly hydrophobic surface of the cells of *P. purpureum* caused by the presence of mucous covering around the cells of the red algae [77]. It was shown that carbon NPs can bind to membranes of microorganisms by hydrophobic interaction and hydrogen bonding formed between surfaces of cells and defect areas of NPs [78]. Hence, the hydrophobic surface of cells might have facilitated adhesion of more hydrophobic carbon NPs to microalgae (Figure 3b,c). Therefore, it could be stated that the red algae *P. purpureum* received higher influence from the samples of carbon NPs.

The current statement correlated with the results of the structural and surface analysis of the samples of carbon NPs (Table 1). It was shown (Figure 1) that the carbon nanotubes sample CNT-2 having the most ordered structure had a lower influence on the growth-rate of *P. purpureum* as compared to the other carbon NPs having unordered structure and a relatively higher hydrophobicity. Therefore, based on inhibition of growth-rate of *P. purpureum* and low influence of carbon NPs (except CNT-2) on esterase activity and membrane potential, we can conclude that the main mechanism of toxic action of these samples on the red microalgae was a physical damage caused by adhesion of clusters of NPs with extracellular mucopolysaccharides of *P. purpureum* [79].

Almost no effect of carbon NPs on growth-rate of *A. ussuriensis*, *H. akashiwo*, and *C. muelleri* was probably caused by the lower mechanical interaction between microalgae cells and the NPs. However, observed esterase activity inhibition, membrane depolarization, and size changes of microalgae cells might be produced by the influence of metal impurities containing in carbon NPs (Table 1). Prior research demonstrated similar effects of metal ions on esterase activity, membranes, and size of microalgae cells [80,81,82].

In general, nanofibers reveal a lower influence on esterase activity and membrane polarization compared to carbon nanotubes. Such a difference is probably caused by the unequal physical accessibility of toxic impurities from different types of NPs to microalgae cells [83].

### 4.2. Silica Nanotubes

The influence of silica nanotubes caused a significant growth-rate inhibition for all species but almost did not change esterase activity, membrane polarization (Table 3, Figure 1), size (Figure 2), and shape of microalgae cells (Figure 4). According to the experimental data and the absence of impurities in the composition of SNT-1 and SNT-2 samples (Table 1), we can conclude that the main toxic mechanism for silica nanotubes was mechanical damage of microalgae cells.

Hydrophilic surface of silica NPs allows them to easily move in the water body [84]. Such properties increase the possibility of NPs to have contact with microalgae cells. Thus, a planktonic species i.e., *C. muelleri*, more frequently had a contact with silica nanotubes and experienced more severe mechanical damage (Figure 1c) compared to benthic laying on the bottom *A. ussuriensis* (Figure 1a), placed near the water surface *H. akashiwo* (Figure 1b), and had defense mucous covering *P. purpureum* (Figure 1d).

Interestingly, the toxic influence of silica nanotubes was reduced with time for all microalgae species except small planktonic algae *C. muelleri*. At the same time, the sample SNT-2 with a larger diameter of nanotubes had higher influence on growth-rate of two microalgae species with larger cells i.e., *A. ussuriensis* and *H. akashiwo*. Previous works showed that the toxicity from the silica nanotubes increases with the increase of microalgae cell diameter [85]. The other research described an increase in the phytotoxic action for silica NPs with an increase of particle size [86]. For *C. muelleri* and *P. purpureum*, which are the algae species having smaller cell sizes, both types of silica nanotubes demonstrated a comparable level of toxicity, though the minor predominance could be seen for the sample SNT-1 (Table 3, Figure 1) with a smaller diameter of nanotubes but a more developed surface area.

In the bioassay on common microalgae *Raphidocelis subcapitata*, the authors claimed the increase of silica nanotubes toxicity with increase of its surface area [84]. Hence, our results were in a good correlation with previous research, and we can conclude that the toxicity of silica NPs strongly depends on their size, surface area, and other surface properties.

However, the cell size changes observed for diatomic algae *A. ussuriensis* under the treatment of silica nanotubes were unexpected and it was in contradiction to the conclusion made for carbon NPs that the size of microalgae changed by the influence of metal ions from particle impurities. Nevertheless, the size of *A. ussuriensis* cells was reduced in the presence of silica nanotubes having no impurities (Table 1, Figure 3a).

Probably, the observed effect could be related to diatomic algae reproduction peculiarities. It is a known fact that the size of the diatomic algae population becomes smaller through the series of cell divisions, and the initial cell size can be kept only by sexual reproduction [87]. Therefore, cell size reduction for *A. ussuriensis* might be caused by a disorder in the reproduction processes. The reason for such disorder is a matter for further investigation and discussion. Moreover, the samples of silica nanotubes did not reveal any influence on the size of the other three microalgae species (Figure 3b–d).

Another interesting observation was the increase in microalgae membrane polarization under the influence of silica NPs (Table 3, Figure 1). Therefore, the mechanisms of toxic action for silica nanotubes cannot depend only on particle diameter and surface area, and future investigations should be focused on the searching of the parameters and their combinations which could influence the toxicity of NPs.

## 5. Conclusions

In this study, the aim was to assess the level of toxicity and the mechanisms of toxic action of carbon nanotubes, carbon nanofibers, and silica nanotubes using four microalgae species as the objects of aquatic toxicity bioassay. The results of this investigation show that (1) carbon nanotubes samples CNT-1 and CNT-2 had non-significant toxic effect on the growth rate of all four microalgae species but caused a high inhibition of esterase activity and depolarization of cell membranes, which was most probably caused by heavy metal impurities in NPs; (2) more hydrophobic carbon NPs with less ordered structure had a higher impact on the red microalgae *P. purpureum* because of higher adherence between the particles and mucous covering of the red algae cells; (3) silica NPs did not affect the esterase activity and membrane potential of the cells of all four microalgae species even at higher concentrations but caused significant inhibition of growth-rate, which indicated predominance of mechanical damage as a mechanism of toxicity for used samples of silica nanotubes.

The findings of this research provide insights for the formation of the principles of safe design, production, and utilization of NPs [88]. We believe that safety ensuring in nanotechnology would be provided only by international cooperation and large-scale nanotoxicology research, including the approaches of bioinformatics, system biology, and other methods of modeling, prediction, and maintaining the handling and interpretation the growing body of research data.

## Figures and Tables

**Table 1:** Characteristics of the nanoparticles used in this study.

Sample	Diameter, nm	Impurities, %	Structure Features
CNT-1	18–20	Fe—0.6; Co—0.3; Al—0.9	Many particles with unordered structure, defect areas with opened ends of a carbon nanotubes
CNT-2		Fe—0.2; Co—0.12; Ca—0.004; Cl—0.08	Ordered structure
CNF-1	90–120	Al_2_O_3_—0.4	Unordered structure, defect areas, the presence of amorphous carbon
CNF-2		Al_2_O_3_—0.4; Ni—3.6	Unordered structure, defect areas
SNT-1	3–4	–	–
SNT-2	45	–	–

* The characteristics given in the table are as given in earlier studies i.e., diameter [47,48], impurities [47], and structural features [41].

**Table 2:** Toxicity assessment criteria and conditions of their registration.

Endpoint	Registration Time	Biomarker	CytoFLEX Emission Channel, nm
Growth-rate inhibition	96 h, 7 days	PI	ECD, 610
Esterase activity	3 h, 24 h	FDA	FITC, 525
Membrane potential	6 h, 24 h	DIOC_6_	FITC, 525
Size	96 h, 7 days	Forward scatter intensity	FSC

**Table 3:** The calculated EC_50_ concentration of growth-rate inhibition, esterase activity inhibition, and membrane depolarization, mg/L.

Sample	Growth-rate		Esterase Activity		Membrane Potential	
	96 h	7 days	3 h	24 h	6 h	24 h
*A. ussuriensis*						
CNT-1	360.0	n/a	29.8 (29.3–30.3)	24.3 (23.9–24.6)	27.4 (26.9–27.8)	23.4 (23.2–23.7)
CNT-2	344.0	560.0	56.2 (54.2–58.3)	42.1 (41.6–52.8)	81.4 (78.2–84.7)	52.7 (51.4–54.1)
CNF-1	>1000	137.6 (135.0–140.2)	94.3 (91.2–97.6)	154.4 (150.3–158.7)	65.4 (62.1–68.8)	41.7 (40.2–43.2)
CNF-2	n/a	170.0 (168.9–171.2)	n/a	297.8 (294.0–301.6)	70.1 (67.4–72.9)	55.5 (55.0–56.1)
SNT-1	>1000	180.1 (173.2–189.1)	n/a	839.0	n/a	97.4. (85.2–111.7)
SNT-2	n/a	95.3 (94.7–95.9)	stimulation 13%–22%*	403.0	176.4 (169.3–183.9)	stimulation 5%–25%*
*H. akashiwo*						
CNT-1	>1000	1000.0	55.1 (53.4–56.4)	38.4 (38.7–40.1)	39.6 (38.9–40.4)	21.6 (21.4–21.7)
CNT-2	>1000	855.0	83.0 (78.9–87.3)	40.9 (40.3–41.5)	65.2 (63.1–67.5)	37.4 (36.4–38.3)
CNF-1	n/a	110.6 (107.5–113.8)	51.2 (50.3–52.0)	82.5 (82.0–83.1)	46.0 (44.4–47.7)	31.4 (30.5–32.2)
CNF-2	n/a	135.8 (133.5–138.2)	stimulation 20%–25%*	168.1 (164.1–172.2)	48.0 (47.0–49.1)	51.9 (51.3–52.4)
SNT-1	>1000	199.1 (190.6–208.2)	532.0	153.9 (148.6–159.5)	256.6 (245.4–268.6)	stimulation 9%–15%*
SNT-2	577.0	57.3 (56.7–57.9)	>1000	193.1 (181.8–205.5)	92.2 (88.6–96.0)	>1000
*C. muelleri*						
CNT-1	313.0	318.0	105.8 (100.5–111.6)	733.0	38.8 (38.0–39.7)	60.9 (58.8–63.1)
CNT-2	>1000	680.0	45.8 (45.1–46.6)	136.5 (131.7–141.5)	60.4 (60.0–60.8)	39.6 (38.9–40.4)
CNF-1	n/a	830.0	59.3 (58.5–60.1)	>1000	39.83 (39.0–40.7)	33.8 (33.2–34.5)
CNF-2	601.0	428.0	stimulation 15%–18%*	stimulation 10%–25%*	60.2 (59.0–61.3)	223.2 (222.5–223.9)
SNT-1	161.4 (158.6–164.3)	59.8 (58.3–61.4)	>1000	n/a	132.6 (127.4–138.1)	159.7 (157.6–161.8)
SNT-2	151.8 (147.9–155.9)	61.1 (58.3–64.1)	107.7 (104.7–110.9)	142.3 (140.8–143.8)	27.9 (27.7–28.2)	74.11 (73.4–74.9)
*P. purpureum*						
CNT-1	28.7 (28.1–29.3)	36.1 (35.2–37)	82.7 (80.9–84.6)	423.0	53.9 (53.4–54.5)	208.6 (202.9–214.5)
CNT-2	178.3 (175.8–180.9)	415.0	16.5 (16.1–16.8)	78.6 (75.7–81.5)	213.2 (209.7–216.8)	86.8 (84.6–89.1)
CNF-1	39.5 (38.3–40.7)	61.5 (60.2–62.8)	n/a	654.0	359.0	stimulation 20%–35%*
CNF-2	106.1 (104.9–107.3)	246.4 (234.7–259.0)	n/a	n/a	692.0	479.0
SNT-1	160.4 (189.7–161.1)	140.5 (137.5––143.7)	stimulation 10%–22%*	n/a	stimulation 42%–45%*	231.4 (229.7–233.1)
SNT-2	285.2 (279.2–291.4)	170.3 (166.8–173.8)	n/a	280.9 (280.2–281.7)	stimulation 82%–90%*	148.0 (110.4–156.1)

95% confidence limits presented in the parentheses; n/a, measured effect was not observed even at the highest concentrations of the sample; *In the cases when influence of NPs caused stimulation of the registered endpoint, the data were represented for concentration of 100 mg/L compared to control.
